# A Machine Learning Approach for Estimating Person Counts Using Anonymous WiFi Data in a University Library

**DOI:** 10.3390/s25227065

**Published:** 2025-11-19

**Authors:** Lucio Hernando-Cánovas, Alejandro S. Martínez-Sala, Juan C. Sánchez-Aarnoutse, Juan J. Alcaraz

**Affiliations:** Department of Information and Communication Technologies, Universidad Politécnica de Cartagena (UPCT), 30202 Cartagena, Spain; lucio.hernando@edu.upct.es (L.H.-C.);

**Keywords:** occupancy, WiFi, machine learning, smart campuses

## Abstract

Accurately estimating indoor occupancy is essential for managing building spaces and infrastructure, with applications ranging from ensuring safe distancing and adequate ventilation during health crises to optimizing energy consumption and resource allocation. However, no existing technology simultaneously achieves accuracy, low-cost, and privacy preservation in indoor occupancy measurement. This study investigates the use of existing WiFi infrastructure as a non-intrusive sensing system, where access points operate as soft sensors that passively collect anonymized connection metadata serving as proxies for human presence. The proposed approach was validated in a university library over eight months, training supervised machine learning regression models on WiFi data and comparing predictions against computer-vision ground truth. The best-performing models (SVR, Ridge, and MLP) consistently achieved R^2^ ≈ 0.95, with mean absolute errors of about 8 persons and relative errors (SMAPE) below 10% at medium-to-high occupancies. Tree-based ensemble models, particularly XGBoost, exhibited weaker generalization at extreme capacity ranges, likely due to data sparsity and sensitivity to hyperparameters. Importantly, no temporal degradation was observed across the 8-month horizon, confirming the long-term stability of the method. Overall, the results demonstrate that WiFi-based occupancy estimation offers a robust, cost-effective, and privacy-preserving solution for real-world deployments.

## 1. Introduction

Smart campuses are emerging as a technological extension of the smart city paradigm, driven by digital transformation and the integration of cyber-physical systems in higher education environments [1,2,3]. Within this framework, university libraries represent critical infrastructures where occupancy monitoring plays a central role in enabling dynamic space allocation, improving user experience, and supporting sustainable operations [4,5].

Indoor people monitoring can be classified into three hierarchical levels [6]: (1) presence, (2) people counting, and (3) individual identification. While presence detection and counting preserve anonymity, identification raises privacy concerns under regulations like the General Data Protection Regulation (GDPR) in the European Union, requiring strict compliance measures. Our work focuses on the counting level as it balances operational utility with privacy preservation.

Building-occupancy monitoring systems employ various methodologies for person counting [7]: (1) environmental/presence sensors (e.g., CO_2_, PIR), which are sensitive to ventilation and room typology, and therefore offer limited spatiotemporal resolution [8,9,10]; (2) computer vision, which—through fine-tuned and well-trained detection models—can achieve very high accuracy in identifying and counting people, making it one of the most precise approaches for this task. However, vision-based systems entail important practical limitations: privacy concerns arise when monitoring public or semi-public spaces, and compliance often requires top-view configurations and edge-processing devices to avoid recording identifiable images. Moreover, deployment and maintenance costs increase rapidly with the number of monitored areas, as each new zone requires additional cameras, processing units, and calibration, which may be infeasible in complex indoor layouts [11,12]; and (3) wireless signals (Wi-Fi/BLE, where BLE stands for Bluetooth Low Energy), which often provide the best trade-off between accuracy, cost, and anonymity. Wi-Fi-based approaches are particularly appealing because most users already carry connected devices, and the existing network infrastructure can be leveraged without installing new sensors. Alternatively, low-cost IoT probes could passively capture wireless signals even from non-associated devices, offering a scalable, privacy-preserving, and economically sustainable solution for large-scale occupancy estimation.

Within wireless approaches, two main strategies can be distinguished: (1) passive detection, which relies on devices acting as sniffers to capture signals such as Wi-Fi probe requests or BLE advertisements. This method does not require users to be connected to the network; however, it demands the deployment of dedicated sensors and specialized hardware, increasing implementation costs and complexity, particularly at large scale. Moreover, most studies assume a one-to-one correspondence between detections and individuals, which can lead to overestimations when a single person carries multiple devices [13,14,15], and (2) leveraging the existing Wi-Fi infrastructure, where APs act as *soft sensors* and provide more stable, persistent telemetry without installing additional sensors, reusing the already-deployed network and reducing deployment and maintenance costs. Nevertheless, Wi-Fi–infrastructure–based methods face key challenges: (1) the difficulty of obtaining reliable ground truth at scale in an automated and sustained manner over time; and (2) the prevalence of short temporal windows (weeks/months) in prior studies, which hinders capturing seasonal behaviors and assessing long-term viability and robustness against temporal drift.

This work analyzes the long-term viability of Wi-Fi telemetry for occupancy estimation. To this end, we build an eight-month labeled dataset (November 2024–June 2025) using a continuous, automated pipeline that integrates institutional Wi-Fi telemetry with a computer-vision system that provides automatic and reliable ground truth. The feature vector is enriched by distinguishing randomized vs. static MAC addresses to infer device class (mobile vs. laptop/desktop) in aggregate, while preserving anonymization and privacy compliance. Our primary goal is to evaluate the temporal robustness of the models (to detect potential performance degradation over time) and to validate their behavior when trained and tested across extended, heterogeneous conditions, confirming stable performance over the course of an academic term. In addition to evaluating the long-term feasibility of Wi-Fi–based occupancy estimation, this work does not focus on a single algorithm but rather on testing several models commonly used in the literature. The objective is to determine which of these approaches performs better overall and which maintain stable performance over time, assessing possible degradation as the dataset extends across several months. For this purpose, we include representative algorithms from different regression families: linear models (Ridge) as a simple and interpretable baseline; margin-based models (Support Vector Regression, SVR), widely adopted for their generalization capacity; tree-ensemble models (Random Forest, ExtraTrees, and XGBoost) for capturing nonlinear relationships; and a simple neural model (Multilayer Perceptron, MLP) to examine the behavior of neural approaches under realistic conditions. This setup allows us to identify which types of algorithms are more suitable for long-term Wi-Fi–based occupancy prediction on large, real-world datasets. Furthermore, we enrich the feature vector by incorporating the number of randomized and static MAC addresses, a distinction not explicitly considered in previous studies. Although its impact in the library environment is limited (since both MAC types appear in similar proportions), it may become informative in more heterogeneous scenarios where the prevalence of randomized MACs varies significantly. However, the proposed framework currently relies on users being associated with access points, which is valid in our library scenario but limits applicability in environments where not all individuals connect to Wi-Fi.

The rest of the paper is organized as follows. Section 2 surveys related work on Wi-Fi–based occupancy estimation and adjacent approaches. Section 3 describes the case study site. Section 4 details the dataset generation pipeline and feature engineering. Section 5 presents the training methodology. Section 6 reports the results, including comprehensive performance metrics and validation against ground-truth occupancy. Finally, Section 7 concludes and outlines directions for future work.

## 2. Related Work

One of the main obstacles in Wi-Fi–infrastructure–based occupancy estimation is obtaining large-scale labeled data, that is, reliable and continuous measurements of true occupancy (ground truth) for training and validating models. Manual on-site counting is costly and unsustainable, which restricts studies to short temporal windows and exposes them to human error.

In response to this challenge, many studies have adopted calibration heuristics based on device-to-person conversion factors. Typically, during a short calibration period (one to two weeks), both headcounts and the number of connected devices per area are recorded, and the average ratio is then used as a multiplier to estimate occupancy thereafter. For example, Ref. [16] estimated campus occupancy at the University of New South Wales by counting Wi-Fi devices over four weeks and applying a fixed factor of 1.3, achieving a correlation of r = 0.85 with observed occupancy. Although pragmatic, this approach fails to capture real-world variability: the device-to-person ratio shifts with the academic calendar (exams, holidays), usage patterns, and other contextual factors. In parallel, some authors have explored capture–recapture methods to infer the number of individuals from observed identifiers [17], with reported Mean Absolute Error (MAE), defined as the average absolute difference between estimated and actual values, ranging from 3 to 40 people depending on the occupancy level. However, applying this approach to Wi-Fi is constrained by MAC address randomization, which introduces time-varying identifiers and detection biases. These effects violate key assumptions of capture–recapture (e.g., persistent and independent “marks”), making consistent estimation difficult. To mitigate the lack of labels, another line of work integrates computer vision as an automated source of ground truth.

The most common systems use top-view cameras with line-based counting to tally entries and exits, synchronizing these data with Wi-Fi telemetry to train supervised models. For instance, Ref. [18] analyzed a small office environment with peak occupancies of 48–74 people over five weeks, categorizing devices by connection time to distinguish permanent and transient users. Their study demonstrated that Wi-Fi connectivity can approximate human presence under controlled conditions. Similarly, Ref. [19] explored a classroom of approximately 80 seats over a single week, correlating Wi-Fi connections and CO_2_ levels with manual headcounts. Their multiple regression model achieved R^2^ = 0.703 when using Wi-Fi data alone and R^2^ = 0.792 when combining Wi-Fi with environmental sensing, confirming the feasibility of Wi-Fi–based occupancy estimation. In another short-term experiment, Ref. [20] monitored a conference room (≈100-person capacity) for six weeks using a commercial EBTRON C100 counter as ground truth. They reported R^2^ values between 0.86 and 0.96 and demonstrated the potential of Wi-Fi telemetry for HVAC optimization. Finally, Ref. [21] conducted one of the most extensive studies to date in a Montreal university library (capacity ≈ 2200). Over nine weeks, they trained regression models—including linear, Random Forest, and Gradient Boosting—using variables such as number of MAC addresses, day, and hour, achieving R^2^ values between 0.92 and 0.96.

Overall, these hybrid Wi-Fi/vision approaches have significantly reduced manual labeling effort and improved ground-truth fidelity. However, most existing studies are short in duration (≈1–2 months) and confined to a single environment, hindering the observation of seasonal phenomena (e.g., start of term, exam periods, holidays) and limiting a comprehensive evaluation of model robustness over time. In this regard, Ref. [4] shows that distinct occupancy patterns can be identified across an academic year, underscoring the need for longer data series to ensure the validity and stability of the estimates.

In contrast, our work provides a comprehensive longitudinal evaluation over eight months (November 2024–June 2025), combining institutional Wi-Fi telemetry with a continuous top-view, line-based computer vision counter as a persistent source of ground truth. This setup enables, for the first time, a long-term synchronization between visual and Wi-Fi data streams. Moreover, we perform a systematic comparison of multiple regression families (linear/margin-based, instance-based, tree-ensemble, and neural) and introduce a detailed temporal performance analysis, including weekly MAE tracking and error segmentation by occupancy level. Together, these elements offer a novel and robust framework for assessing Wi-Fi–based occupancy estimation in real-world conditions.

The main contributions of this study are as follows:

- Longitudinal evaluation: We conduct an eight-month study (November 2024–June 2025) spanning multiple seasonal regimes, providing a broader temporal scope than prior short-term evaluations.
- Methodological design: We leverage institutional Wi-Fi telemetry combined with a top-view, line-based computer vision counter as continuous ground truth, and enforce a strict non-overlapping split (four months for training, four months for testing) without intermediate retraining, to emulate real production conditions.
- Detailed performance analysis: Beyond reporting global metrics, we assess temporal robustness by tracking weekly MAE to detect performance drift and analyze errors by occupancy level, covering low, medium, and high loads.
- Comparative modeling study: We benchmark multiple regression families (including linear/margin-based, instance-based, tree ensembles, and neural models) to identify the most suitable approaches for the occupancy estimation problem.
- Feature vector design: We enrich the feature set by incorporating the number of randomized and static MAC addresses (a distinction not explicitly considered in previous studies) to better capture device-type diversity and support future deployment in heterogeneous environments.

## 3. Methodology and Case Study

The case study focuses on the Antigones Library, located within the School of Telecommunications Engineering building. It is the main library of the Universidad Politécnica de Cartagena (UPCT). The library is equipped with a top-view, line-based counting system at its main entrance, which provides an automated and continuous ground truth of occupancy with high accuracy. This setup enables sustained data generation over time and supports the supervised validation of occupancy-estimation models using logs from the existing Wi-Fi infrastructure.

### 3.1. Process Framework: CRISP-ML(Q)

We follow the CRISP-ML(Q) process model to structure the study and embed quality assurance [22]. The framework comprises business and data understanding, data engineering, model engineering, evaluation, deployment, and monitoring and maintenance. In this feasibility study, only the first four phases were instantiated whereas deployment and monitoring were emulated offline by training once and evaluating on a four-month holdout with weekly MAE and drift analyses. The subsequent sections cover each phase in context: Section 3.2, Section 3.3 and Section 3.4 (understanding and data sources), Section 4 (data engineering), Section 5 (model engineering), and Section 6 (models’ evaluation).

### 3.2. Study Area

The library is a university facility covering approximately 1900 m^2^, with a maximum capacity of 270 people. Inside the building, seven Wi-Fi access points (APs) are deployed; these APs act as soft sensors, providing counts and telemetry of the devices connected within the facility.

Operationally, the library is open Monday–Friday, 08:00–21:30 (closed on weekends). The analysis period spans November 2024 to June 2025, which yields observations across low (vacation), medium (regular term), and high (exam periods) occupancy regimes.

Assumptions for this environment:

- Seasonality and prolonged user stays. As a study space, users typically remain for extended intervals, leading to relatively stable occupancy over time and persistent Wi-Fi sessions across sampling intervals.
- Multiple devices per user. A single user may carry 0, 1, 2, or more devices (e.g., smartphone, laptop, tablet). This non-one-to-one device-to-person relationship adds complexity to occupancy estimation (the ultimate target), which we address through feature engineering and modeling.

### 3.3. Wi-Fi Infrastructure

**Figure 1:**
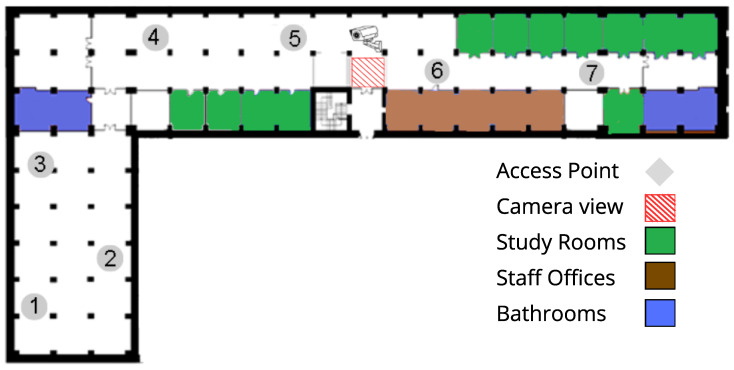
Spatial layout of access points within the university library.

**Figure 2:**
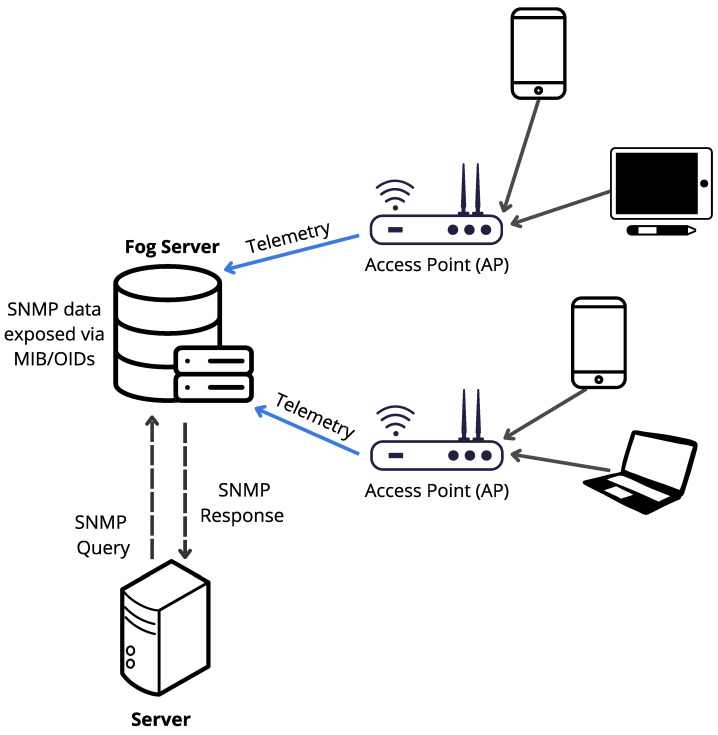
Data flow in the Wi-Fi infrastructure.

As shown in Figure 1, the library contains seven Wi-Fi access points (APs) distributed across the floor area. In addition to providing wireless coverage, these APs act as soft sensors: they record signals from associated devices that serve as proxies to estimate occupancy. All telemetry is centralized by a controller (hereafter, Fog Server), which stores the data reported by the APs and exposes it via SNMP (Simple Network Management Protocol) through its MIB (Management Information Base). Each metric is available as an object identified by an OID (Object Identifier). This design enables remote querying of the variables of interest without needing direct access to each individual AP (see Figure 2).

### 3.4. Computer-Vision System

**Figure 3:**
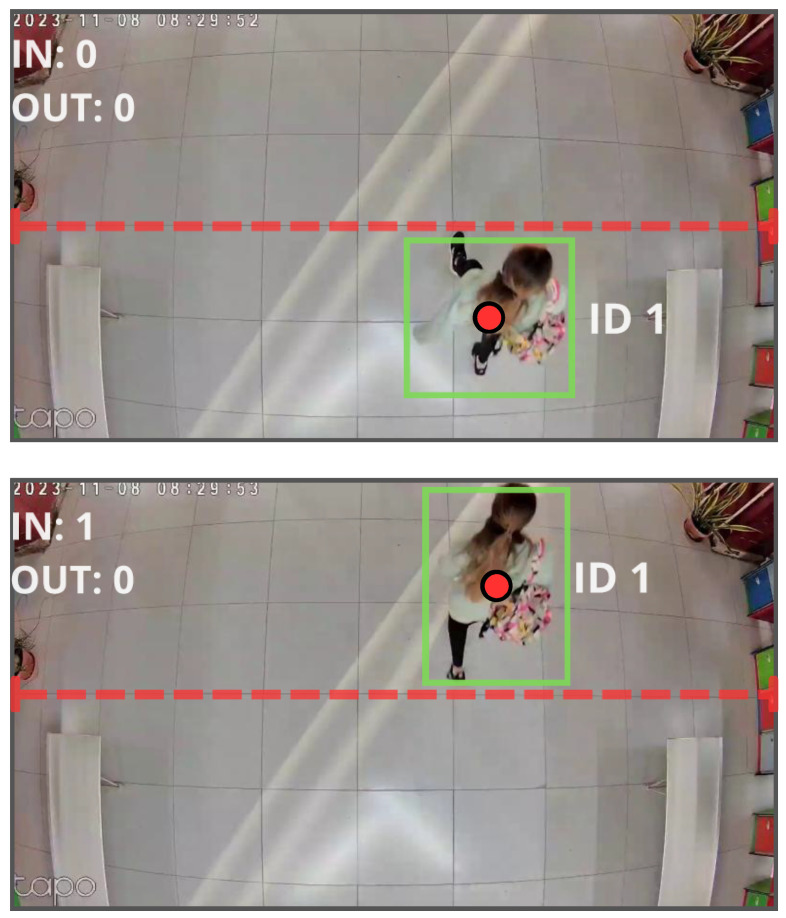
Example of automated entry/exit detection.

The library employs a line-based counting system installed in the entrance corridor. The method defines a virtual line on the top-view image and counts entries and exits according to the crossing direction. From these events, the system integrates the instantaneous occupancy of the room, producing an automated and continuous ground truth during operating hours (see Figure 3).

The detector is based on a YOLO person detector coupled with a tracking algorithm, which preserves object identity while crossing the line. In internal validations, the system achieves >94% accuracy in counting entries and exits [23]. The system exposes a REST API that provides per-minute occupancy (timestamp and cumulative occupancy) for a given day, enabling automated ground-truth collection.

## 4. Dataset Generation

### 4.1. Features Selection

As part of the exploratory data analysis (EDA), we audited 18 telemetry fields exposed by the Fog Server via SNMP and evaluated them under four criteria: (i) proxy relevance for occupancy, (ii) data quality, measured by missingness and temporal stability across access points and time slots, (iii) portability across sites, avoiding highly site-specific fields, and (iv) privacy compliance under GDPR.

We keep a minimal, high-quality set that serves as robust proxies while preserving privacy: (1) device MAC address, (2) associated AP IP, (3) bytes transmitted per device, (4) bytes received per device and (5) association uptime.

EDA indicated that several fields fail at least one criterion:

- Operating system: In more than 40% of the records, the value of this field appeared as “unknown.” This occurs because the Fog Server attempts to infer the operating system from device network fingerprints, although the exact inference mechanism is not documented, and in most cases, it simply fails to identify the OS. Given both the high proportion of missing or unknown values and the lack of transparency about how this field is derived, and was therefore excluded from the feature set.
- UserDeviceIP: The IP address of each connected device was also discarded, since it does not provide any additional information beyond what is already captured by the MAC address. The MAC alone is sufficient to identify unique devices for counting purposes, making the IP redundant for this task.
- Human-readable names or labels: These fields were potentially identifying and were therefore excluded to preserve anonymization.
- WLAN ID and related configuration fields: These parameters were excluded because they are specific to the internal configuration of the university’s Wi-Fi network, which would prevent the model from generalizing to other campuses or environments. Their inclusion would introduce dependencies tied to local network topology rather than user behavior.
- Redundant traffic transformations: Alternative encodings of the same byte counters that added no new signal were removed.
- PacketRetryCount: Metrics related to packet retransmissions or retry counts were discarded because their variation is mainly driven by channel conditions, signal interference, or temporary congestion, rather than by the number of people present. As such, these fields do not reflect occupancy dynamics and were not included in the final feature set.
- Constant or non-discriminative fields: For example, link type (wired versus Wi-Fi), when all sessions correspond to Wi-Fi connections (see Table 1).

### 4.2. Feature Engineering

**Figure 4:**
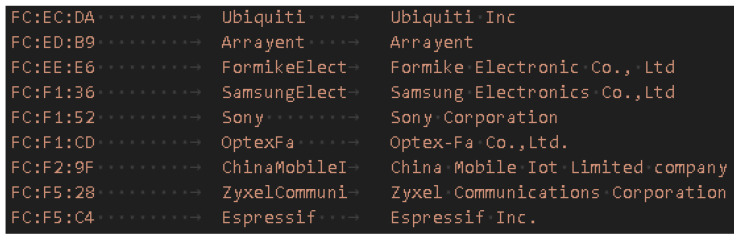
Extract from the Wireshark Manufacturer Database.

To make the collected information suitable for predictive modeling, we apply a transformation and enrichment process. In particular, the MAC addresses exposed by the controller allow us to infer indirect information about the device type. To this end, each MAC is classified into one of two mutually exclusive categories:

- Randomized MACs: addresses generated dynamically by the operating system to preserve privacy. These addresses change frequently and are identified because the second hexadecimal digit of the first byte takes the value 2, 6, A, or E (i.e., the locally administered bit is set).
- Static MACs: globally unique addresses assigned by the manufacturer that retain a valid OUI (Organizationally Unique Identifier). Classification was performed by matching the first three bytes against a manufacturer database (e.g., the Wireshark Manufacturer Database) (see Figure 4).

### 4.3. Data Pipeline Architecture: Wi-Fi Ingestion and Feature-Vector Construction

#### 4.3.1. Module I—Periodic Telemetry Ingestion (Historical)

**Figure 5:**
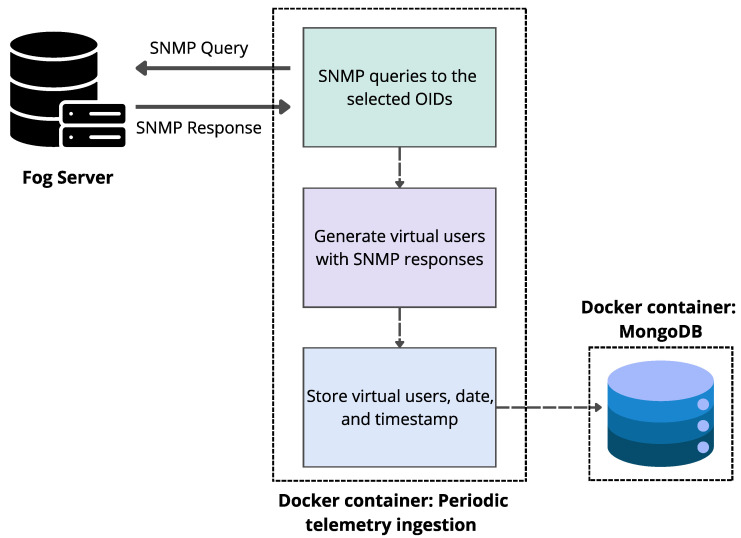
Periodic telemetry ingestion pipeline.

This module issues periodic SNMP queries to the Fog Server to build a history of associated devices and the selected variables. In each cycle, the defined OIDs are queried and telemetry data are consolidated by linking all variables associated with the same device (MAC, AP IP, transmitted bytes, received bytes, and association uptime) to obtain a unified per-device view at each interval. The resulting snapshot is stored in the database with its timestamp. A 3-min cadence is used to complete queries and writes without overlapping, ensuring regular sampling over time (see Figure 5).

#### 4.3.2. Module II—Feature-Vector Generation

**Figure 6:**
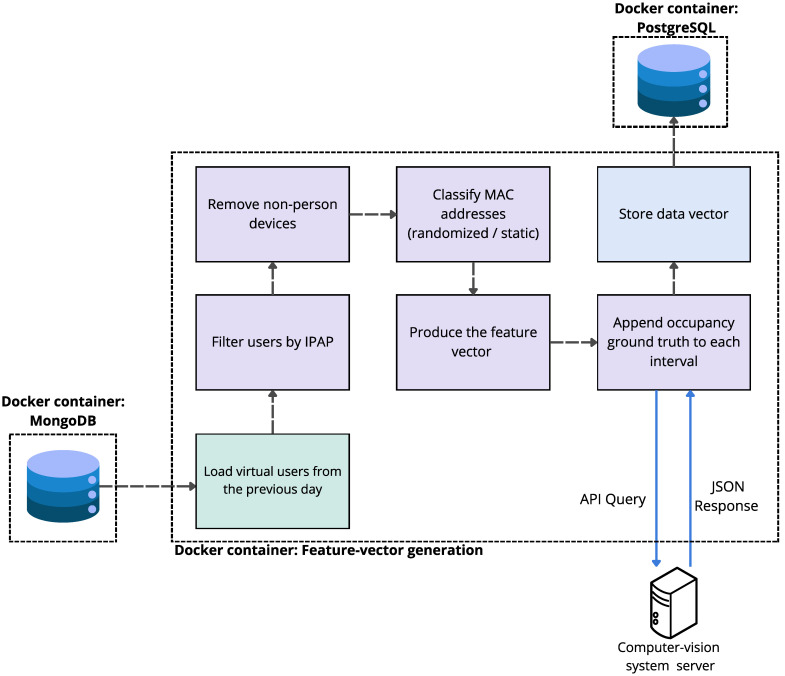
Feature-vector generation pipeline.

Once per day, using the previous day’s history, we generate the data (feature) vector that describes the library’s state at each 3-min timestamp. First, all ingestion records from the previous day are loaded; then they are filtered by AP to restrict the scope to the study area. Next, non-person devices are pruned, removing activity outside opening hours (overnight presence). On the resulting set we apply feature engineering, including MAC classification (randomized vs. static), and produce the feature vector for each time interval. Finally, the REST API of the computer-vision system was queried to append the corresponding occupancy (ground truth) to each interval (see Figure 6).

### 4.4. Feature Vector

The pipeline in Section 4.3.2 produces a structured, consistent, and representative dataset to feed occupancy-prediction models on campus spaces. Each record captures the aggregate state of the study area at a 3-min timestamp, combining features derived from Wi-Fi telemetry with the target variable (ground-truth occupancy from the vision system, see Table 2).

**Figure 7:**
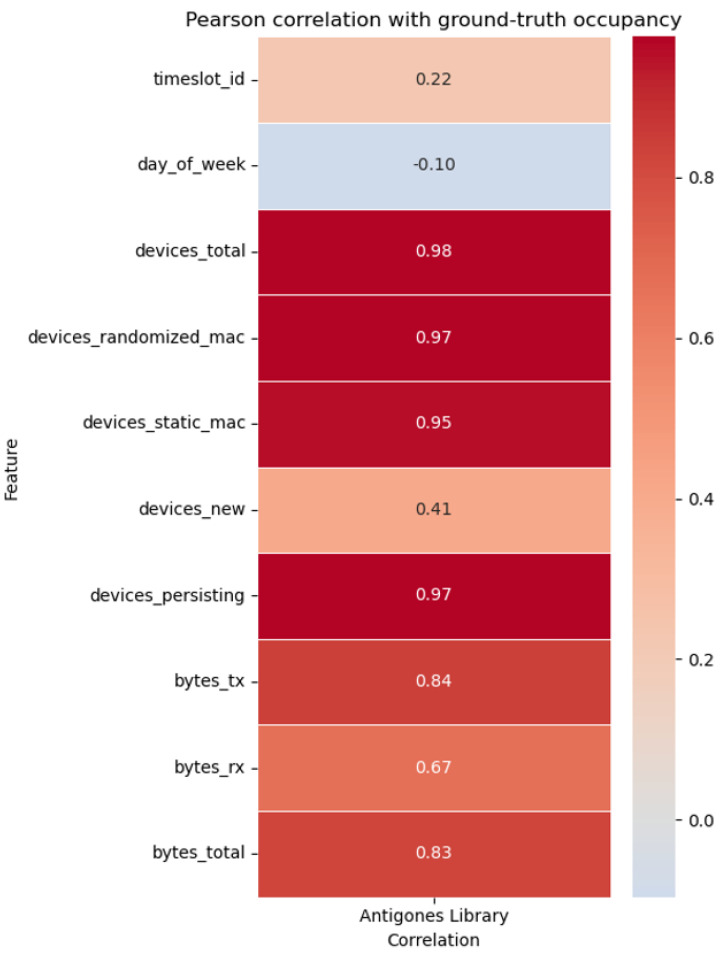
Pearson Correlation of Telemetry Features with Ground-Truth Occupancy.

To further assess the representativeness of the feature vector, we computed Pearson correlations between each candidate feature and the ground-truth occupancy. As shown in Figure 7, device-based features exhibit the highest correlation values (≈0.95–0.98), while traffic-based variables (bytes transmitted/received) also correlate positively (≈0.67–0.84).

Although features such as *devices_total*, *devices_randomized_mac*, and *devices_static_mac* show very high mutual correlation, we decided to keep them in the final dataset. In the library environment, both randomized and static MACs appear in nearly equal proportions (around 50%), resulting in similar performance even when one of them is removed. However, in other campus areas, such as offices, this balance differs markedly. Retaining these features ensures consistency and allows for reliable comparisons across heterogeneous environments in the future.

### 4.5. Temporal Scope of the Dataset

The dataset was built from eight months of data (November to late June) and split into two non-overlapping temporal blocks to ensure diversity and robustness:

- Training set: 86 days from November 2024 to February 2025, used to train the models.
- Test set: 91 days from March 2025 to June 2025, used to simulate production conditions without intermediate retraining.

Both periods include heterogeneous usage scenarios (low-activity days, typical term days, and exam-period peaks) allowing performance to be assessed across varied contexts and reducing bias tied to specific days or situations. Days when the vision system’s cameras were inoperative were excluded, as the ground-truth occupancy label could not be guaranteed.

## 5. Training Methodology

This study evaluates seven algorithms spanning complementary families: a linear model (Ridge), an instance-based method (KNeighborsRegressor), a margin-based model (Support Vector Regression, SVR), three tree ensembles (RandomForestRegressor, ExtraTreesRegressor, XGBRegressor), and a multilayer perceptron (MLP). This selection contrasts different inductive biases to identify which approach best fits occupancy estimation from Wi-Fi telemetry.

Preprocessing procedure was identical across models and implemented with pipelines to prevent data leakage. The categorical variables *day_of_week* and *timeslot_id* were encoded via one-hot encoding, while the continuous variables *bytes_tx* and *bytes_rx* were standardized (zero mean, unit variance) to stabilize scale-sensitive algorithms such as KNN, SVR, and MLP. All transformations were fitted exclusively on the training folds and then applied to their corresponding validation folds.

Training used 86 days of data spanning November 2024 to February 2025. For each algorithm, we performed hyperparameter optimization with Optuna, using stratified 5-fold cross-validation. In this context, the stratification variable was *timeslot_id*, which groups samples by their timestamp. Stratification ensures that each fold contains a representative distribution of these time slots, so that training and validation sets reflect similar temporal patterns. This is important because occupancy strongly depends on the time of day (e.g., morning vs. evening), and a naive split could lead to hour-of-day bias, where the model is trained predominantly on one set of hours but evaluated on another, artificially inflating or deflating performance. By preserving the temporal distribution of time slots across folds, we obtain a fairer assessment of model generalization.

The objective function was the root-mean squared error (RMSE), defined as the square root of the average of squared differences between predicted and actual values, which penalizes larger errors more heavily than MAE. The mean RMSE across folds was minimized to select the best hyperparameters for each model, after which the final model was retrained on the full training set (see Table 3).

## 6. Results

After training the models with four months of data (November 2024–February 2025), we evaluated their performance on an independent 91-day test set (March–June 2025) covering low, medium, and high occupancy regimes.

### 6.1. Metrics (Mar–Jun 2025)

For each model, we report metrics over the four test months:

- MAE (Mean Absolute Error): average error in number of people; indicates how many people, on average, the predictions deviate from the true occupancy.
- RMSE (Root Mean Squared Error): also an average error measure but penalizes large errors more strongly, making it sensitive to peaks and outliers.
- SMAPE (Symmetric Mean Absolute Percentage Error): a variation of the Mean Absolute Percentage Error (MAPE), which computes the average percentage difference between predicted and actual values. While MAPE is widely used due to its interpretability, it becomes unstable when actual values are close to zero. SMAPE addresses this issue by symmetrizing the denominator, making it more robust for low or near-zero occupancies and therefore more suitable for occupancy estimation.
- $R^{2}$ (coefficient of determination): fraction of the variance of true occupancy explained by the model relative to a baseline that predicts the test-set mean. In our context, we interpret $R^{2}$ as the model’s ability to track day-to-day rises and falls in occupancy. A high $R^{2}\;$ indicates good tracking of the temporal shape; $R^{2}\;$ ≈ 0 suggests no improvement over the mean; a negative $R^{2}\;$ indicates performance worse than that baseline (see Table 4).

Overall, the models achieved competitive metrics over an extended validation horizon. SVR delivers the best overall performance (lowest RMSE/MAE and the lowest SMAPE, with $R^{2}\;$ = 0.955). Ridge and MLP are very close, also with high $R^{2}\;$ ≈ 0.95. KNN, Random Forest, and Extra Trees form a second tier with similar results. XGBoost lags behind in this setting (higher RMSE and MAE, $R^{2}\;$ = 0.907). In summary, linear and margin-based approaches and MLP tend to perform slightly better, whereas tree ensembles show somewhat lower performance on this dataset.

### 6.2. Error by Occupancy Range

**Figure 8:**
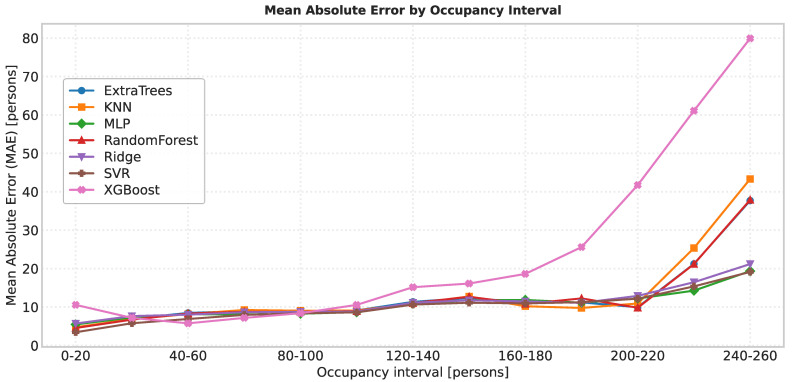
Mean Absolute Error (MAE) by occupancy interval.

Figure 8 reports the Mean Absolute Error (MAE) across occupancy ranges, grouped in 20-person bins (from 0–20 up to 240–260). As expected, the absolute error increases with the number of users in the room, since prediction deviations naturally accumulate at higher occupancies. Three distinct patterns were observed:

1. XGBoost shows the steepest error growth. This behavior may be attributed to its high sensitivity to hyperparameter tuning; the chosen configuration may not be optimal for this dataset, or the model may overfit medium ranges and generalize poorly in the extremes.
2. ExtraTrees, KNN, and Random Forest (tree-based families) perform comparably well at low and medium ranges but deteriorate noticeably at high occupancies. This is likely explained by the scarcity of training samples in those ranges: high occupancy situations are less frequent and occur only on specific peak days.
3. SVR, Ridge, and MLP achieve the most robust performance at high occupancies. Their linear (or margin-based) components allow them to extrapolate more gracefully when training data is sparse, making them less dependent on having many examples of extreme scenarios.

**Figure 9:**
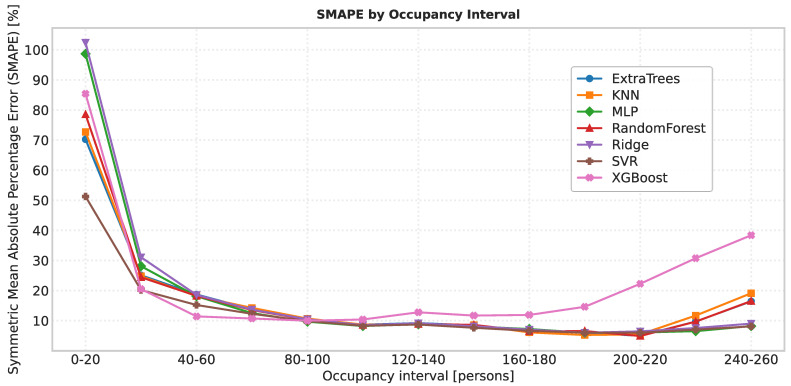
Symmetric Mean Absolute Percentage Error (SMAPE) by occupancy interval.

Figure 9 shows the Symmetric Mean Absolute Percentage Error (SMAPE) for the same occupancy ranges. Again, the three model families emerge. At very low occupancies, the relative error is high, in some cases approaching 100%. This is due to the variability in the device–person ratio: with only a few people in the room, a single individual carrying multiple devices (or none at all) can heavily distort the signal. Nevertheless, SVR already achieves relatively lower SMAPE values (≈50%), compared to 70–100% for the other models. As occupancy increases beyond ≈40 persons, the relative error rapidly stabilizes around 10% or lower for SVR, Ridge, and MLP, while tree-based ensembles degrade, and XGBoost shows the poorest behavior at the upper extremes. Overall, the analysis indicates that linear and margin-based models and MLP are more resilient to data scarcity at high occupancy, whereas tree ensembles are more dependent on sufficient training samples and prone to error amplification in rare extreme cases.

### 6.3. Temporal Stability (Weekly MAE)

**Figure 10:**
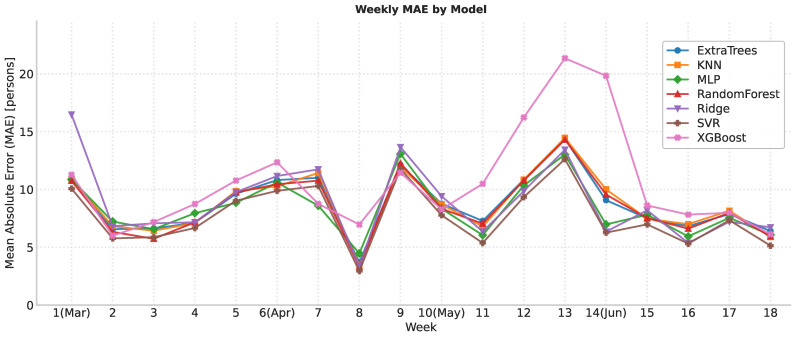
Weekly trend of mean absolute error (MAE).

To assess whether the models degrade over time (whether performance worsens as we move away from the training period), we aggregate errors by calendar week and plot the weekly MAE across the four test months. This aggregation reduces day-to-day noise and makes trends easier to spot. As shown in Figure 10, there is no upward trend: the error remains around 8–10 people on average, with isolated peaks corresponding to specific situations. Consequently, we find no evidence of systematic degradation during March–June. Consistent with previous analyses, SVR, Ridge, and MLP achieve the lowest and most stable weekly MAE, while XGBoost stands out as the least robust, particularly weeks with higher occupancy.

To further assess temporal robustness, shorter training windows of one and two months were also tested, keeping the remaining period for validation. The results showed a slight overall increase in MAE (below one person on average) but maintained the same weekly trend, confirming that model performance remains temporally stable even with reduced training durations and that no significant concept drift occurs throughout the evaluation period.

## 7. Discussion

This study presented a comparison of machine learning models for occupancy estimation from Wi-Fi telemetry, supported by an end-to-end automated pipeline that produced eight months of labeled data. The infrastructure enabled training on four months (Nov–Feb) and validation on an additional four months (Mar–Jun), thus significantly extending the evaluation horizon relative to prior work.

The results confirmed competitive performance on the test set. As expected, absolute error (MAE) increases with occupancy, while relative error (SMAPE) stabilizes at around 10% for medium and high ranges. The main challenge arises at low occupancies, where the small number of people combined with the variable number of devices per person (from none to several) introduces high variability, making percentage-based metrics less reliable in these conditions.

Across model families, Ridge, SVR, and MLP consistently achieve the best performance, maintaining robustness across all occupancy levels. Their linear or margin-based nature enables them to extrapolate more effectively in rarely observed scenarios such as high-occupancy peaks, where tree ensembles (Random Forest, ExtraTrees, XGBoost) tend to degrade. Importantly, no temporal degradation was observed during the four-month validation period: MAE fluctuations reflected week-to-week variations in library usage (low vs. high occupancy days) rather than a systematic decline in model accuracy.

As future work, we plan to extend the validation horizon to longer periods (ideally a full year) to further confirm stability, and to evaluate the approach in additional campus areas and buildings with different capacity ranges and usage patterns (e.g., laboratories, offices, and common spaces). This will enable assessment of the system’s generalization under more diverse behavioral and connectivity conditions, including smaller venues where SMAPE challenges are most pronounced. Further work will investigate prediction intervals to strengthen operational decision-making. Additionally, alternative encoding strategies for temporal features such as *timeslot_id* and *day_of_week* will be explored to better capture non-linear and cyclic occupancy patterns across diverse environments. Moreover, the current limitation of detecting only associated devices will be addressed by integrating passive sensing mechanisms (e.g., probe, FTM, and NDP frames) to include non-associated users and enhance the system’s applicability across heterogeneous populations with distinct mobility and connectivity behaviors.

## Figures and Tables

**Table 1:** Selected Telemetry Features: Usage and Rationale.

Telemetry Field (Example)	Used	Rationale
Device MAC	Yes	Aggregate counts and randomized vs. static split; raw MAC not modeled
Associated AP IP	Yes	AP-level aggregation and device–AP association
Bytes TX/Bytes RX	Yes	Direct activity proxies with low missingness
Association uptime	Yes	Session persistence proxy
OS type	No	>40% unknown; unclear derivation
Device IP	No	Redundant for counting; privacy exposure
Human-readable name/label	No	Privacy-sensitive
Wlan ID	No	Site-specific; low portability
Packet retries (TX/RX)	No	RF-driven noise; weak proxy
Alternative traffic formats	No	Redundant transformations

**Table 2:** Feature vector.

Variable	Type	Description
day_of_week	Categorical	Day of week (1 = Monday, …, 7 = Sunday).
timeslot_id	Categorical	Numeric ID of the 3-min time slot within the day (e.g., 0: 08:00; 1: 08:03; …).
devices_total	Numeric	Total number of unique devices detected.
devices_randomized_mac	Numeric	Number of devices with randomized MAC.
devices_static_mac	Numeric	Number of devices with static MAC (valid OUI).
devices_persisting	Numeric	Devices present in both the previous and current interval (t − 1 and t).
devices_new	Numeric	Devices present in t but not in t − 1.
bytes_tx	Numeric	Bytes transmitted during the interval.
bytes_rx	Numeric	Bytes received during the interval.
bytes_total	Numeric	Sum of transmitted and received bytes.

**Table 3:** Hyperparameter Search Space for Each Model.

Model	Hyperparameter	Search Type/Range
SVR	C	Float in [1 × 10^−4^,1]
	epsilon	Float in [1 × 10^−5^,5]
	kernel	Categorical: linear, poly, rbf}
Ridge	alpha	Float in [1 × 10^−4^,1]
Random Forest	n_estimators	Integer in [10,50]
	max_depth	Integer in [5,30]
XGBoost	n_estimators	Integer in [3,25]
	max_depth	Integer in [2,30]
	learning_rate	Float in [0.01,0.1]
	subsample	Float in [0.2,0.6]
	colsample_bytree	Float in [0.5,1.0]
Extra Trees	n_estimators	Integer in [10,100]
	max_depth	Integer in [5,30]
	min_samples_split	Integer in [2,10]
	min_samples_leaf	Integer in [1,5]
KNN	n_neighbors	Integer in [3,30]
	weights	Categorical: uniform, distance}

**Table 4:** Performance Comparison of Models on the Test Set.

Model	RMSE	MAE	SMAPE	R^2^
Ridge	10.367	8.068	19.34	0.953
KNN	11.451	8.781	18.96	0.943
SVR	10.178	7.775	15.29	0.955
Random Forest	11.181	8.645	18.36	0.946
Extra Trees	11.393	8.790	18.85	0.944
XGBoost	14.696	10.508	19.21	0.907
MLP	10.64	8.40	19.98	0.95

## Data Availability

The data in this study are available from the corresponding author upon request.
